# Data-Driven Optimal Assistance Control of a Lower Limb Exoskeleton for Hemiplegic Patients

**DOI:** 10.3389/fnbot.2020.00037

**Published:** 2020-07-03

**Authors:** Zhinan Peng, Rui Luo, Rui Huang, Tengbo Yu, Jiangping Hu, Kecheng Shi, Hong Cheng

**Affiliations:** ^1^School of Automation Engineering, University of Electronic Science and Technology of China, Chengdu, China; ^2^Department of Sports Medicine, The Affiliated Hospital of Qingdao University, Qingdao, China

**Keywords:** walking assistance control, reinforcement learning, leader-follower multi-agent system, lower limb exoskeleton, hemiplegic patients, actor-critic neural network

## Abstract

More recently, lower limb exoskeletons (LLE) have gained considerable interests in strength augmentation, rehabilitation, and walking assistance scenarios. For walking assistance, the LLE is expected to control the affected leg to track the unaffected leg's motion naturally. A critical issue in this scenario is that the exoskeleton system needs to deal with unpredictable disturbance from the patient, and the controller has the ability to adapt to different wearers. To this end, a novel data-driven optimal control (DDOC) strategy is proposed to adapt different hemiplegic patients with unpredictable disturbances. The interaction relation between two lower limbs of LLE and the leg of patient's unaffected side are modeled in the context of leader-follower framework. Then, the walking assistance control problem is transformed into an optimal control problem. A policy iteration (PI) algorithm is utilized to obtain the optimal controller. To improve the online adaptation to different patients, an actor-critic neural network (AC/NN) structure of the reinforcement learning (RL) is employed to learn the optimal controller on the basis of PI algorithm. Finally, experiments both on a simulation environment and a real LLE system are conducted to verify the effectiveness of the proposed walking assistance control method.

## 1. Introduction

With the increasing requirement of accomplishing complex or difficult tasks in the fields of industry and human daily life, wearable devices/robots have attracted more attentions (Fang et al., 2018, 2019). As one of wearable devices, the lower limb exoskeleton (LLE) integrates artificial intelligence technologies, control and robotic theory, and has become a hot topic own to its practical applications. Note that so many injuries caused by neuromuscular diseases, and accidents reduce the walking ability, most assistive exoskeletons are developed to aid paraplegic patients (Dollar and Herr, 2008; Strausser and Kazerooni, 2011). On the other hand, stroke has gradually become a global health-care problem, which inspires many researchers to pay attention to walking assistance or rehabilitation case for hemiplegic individuals (Ho et al., 2011; Bortole et al., 2015; Iqbal and Baizid, 2015; Louie and Eng, 2016).

From the functions point of views, the LLE can be categorized in three classes of applications, namely, strength augmentation (Walsh et al., 2006; Huang et al., 2016, 2019), walking assistance (Esquenazi et al., 2017; Zhang et al., 2017), and rehabilitation (Sankai, 2010; Huo et al., 2014). For strength augmentation, the wearers usually have walking ability, and the influence of human-robot interaction force should be considered in the controller designs. For walking assistance case, one usually uses the LLE to assist patients' walking/training in which the patients lose their ability to walk. Thus, LLE has served as a device for rehabilitation/walking training with paraplegia and hemiplegia. In recent, some researchers have introduced biological signals of human body into the controller designs, such as Electromyography signal (EMG) (Kiguchi et al., 2004) and Electroencephalogram signal (EEG) (Kilicarslan et al., 2013).

In the early research works of rehabilitation and gait recovery of hemiplegia, researcher proposed Ankle-Foot Orthosis (AFO) to achieve good recovery performance (Tyson and Thornton, 2001; Fatone et al., 2009). In order to provide active power assistance for hemiplegic patients, many powered orthosis with active motors have been developed, such as active AFO developed by Blaya and Herr (2004) and Series Elastic Remote Knee Actuator (SERKA) developed by Sulzer et al. (2009). However, these kinds of orthosis are designed for repairing local motion function of hemiplegic patients in particular scenarios, such as the SERKA is design for stroke patient with stiff-knee gait (SKG).

For the assistance control problem of LLE with hemiplegia, one usually focus on how to derive the LLE to generate a normal motion that aid the patients walking or recovering (Maciejasz et al., 2014; Hassan et al., 2018). Sankai developed a single leg exoskeleton system for hemiplegic patients based on the Hybrid Assistive Limb (HAL) (Kawamoto et al., 2009). For the studies on the HAL system with single leg case, motion information of the unaffected side is generated to synchronize gait of the affected side (Kawamoto et al., 2014). Note that the single leg based HAL system should be re-designed as the wearer has different disabled side. In Fisher et al. (2011), a powered exoskeleton was used to improve patients with hemiparesis walking function via robot assisted gait training. In Murray et al. (2014), the authors proposed a control approach of a LLE to provide walking assistance, without giving desired joint angle trajectory, for facilitating recovery. More recently, the walking assistance control problem for a LLE with hemiplegia was investigated via a learning-based control method (Huang et al., 2018).

In most of the existing relevant works, the case of disturbances caused by system or external environment has not been taken into consideration in the designs of controllers. In fact, disturbances caused by system or external environment will affect the control performance of system, which should be considered in controller designs. On the other hand, the precise system dynamics of exoskeleton is difficult to establish, which decreases the control performance of the *model-based* methods in real systems. To solve this issue, the system identification is needed that would introduce new approximation errors. Therefore, the motivation of this paper aims to address these problems.

Motivated by the above-mentioned discussions and observations. In this paper, a data-driven optimal control (DDOC) strategy is proposed for walking assistance of lower exoskeleton with hemiplegic patients. First, the interaction communications between the both two low limbs of LLE and hemiplegic patient are modeled as a *leader-follower multi-agent system* (LFMAS) framework. Then, a policy iteration (PI) algorithm is employed to compute the optimal assistance controller. Further, in order to improve adaptive performance for walking assistance with different hemiplegic patients, a RL method, called actor-critic neural network (AC/NN), is proposed to achieve better control performance, where the learning process only relays on measurement data from the LLE system. The main contributions of this paper can be summarized as follows:

1. Different from most of the existing control method which is designed in a model-based fashion, a DDOC strategy based on PI algorithm is proposed to learn the optimal assistance controller for walking. The proposed method is designed in a model-free manner without the requirement of the complete knowledge about the accurate dynamics of the exoskeleton system and system identification.
2. An adaptive online-learning based AC/NN structure is employed for the implementation of the controller design, which aims to perform adaptability performance for different patients and achieve good robust against disturbances.

Moreover, the proposed DDOC method is validated through a two degree-of-freedom (2-DOF) simulation environment, and then it is successfully applied on a real LLE system with healthy subjects who simulate paraplegia. Both simulation and experimental results verify that the proposed control approach has robustness performance against disturbances and has adaptive ability for different wearers or even the same wearer with different gait patterns.

The rest of this paper is organized as follows. In section 2, the modeling process of LFMAS for exoskeleton system with hemiplegic patients is established, the system dynamics of the exoskeleton and problem formulation are given. Then, section 3 proposes the PI based optimal assistance controller designs. Section 4 proposes the data-driven adaptive control strategy by making using of RL framework on the basis of the PI algorithm. In section 5, the proposed control methods are illustrated in simulation scenario and is applied to an actual exoskeleton system with healthy people who simulate hemiplegic patients in section 6. Section 7 gives the conclusions and future work.

## 2. Modeling and Problem Formulation

In this section, the modeling process for the LLE with hemiplegic patients, namely LFMAS, is given to describe the interaction relations among both lower limbs of LLE and patients' legs. An information exchange rule is introduced for the LFMAS. Then, the system dynamics and control problem are formulated.

### 2.1. Modeling Exoskeleton System as LFMAS

In this paper, the focus is aim at designing an adaptive assistance controller of a LLE system with both lower extremities to assist hemiplegic individual walking. It should be noted that, for hemiplegic patients, one of the two legs usually loses walking ability and the other one is normal. Therefore, before introducing the controller designs, it is necessary to tackle how to model the interaction relations among them appropriately such that both low limbs and the LLE can achieve their mutual communication.

**Figure 1:**
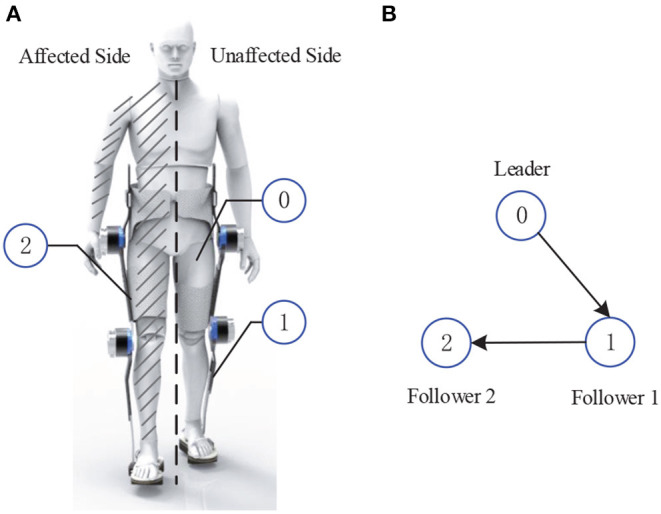
The modeling of Leader-Follower Multi-Agent System. **(A)** The schematic diagram of the LFMAS (The notations 0, 1, and 2 denote leader, follower 1, and follower 2, respectively). **(B)** Communication topology network of the LFMAS structure.

In light of the cooperative distributed control, *leader-follower* mechanism has been wildly utilized in multi-agent systems control (Hu and Feng, 2010), where the main idea is that information interactions among agents are achieved in a distributed way. In this paper, this mechanism is extended to model the unaffected leg of the hemiplegic patients and the both lower extremities of exoskeleton as a LFMAS, where the structure of the LFMAS for exoskeleton system with hemiplegic individuals is illustrated in Figure 1A. That is, the exoskeleton with hemiplegia is divided into three components: one leader agent and two follower agents. In other words, the unaffected leg of patient is regarded as the leader of LFMAS, equipped with an Inertial Measurement Unit (IMU) sensors for measuring its joints' states. Furthermore, both two lower extremities of the LLE system are defined as two follower agents, i.e., follower 1 and follower 2 which can be described as follows:

1. *Follower* 1 is the exoskeleton leg of unaffected side, which synchronizes the leader agent's (unaffected side of patient's leg) motion immediately.
2. *Follower* 2 is the other side of exoskeleton's limb with the disabled leg of patient, the patient's disabled leg is tightly connected with the exoskeleton.

In the framework of LFMAS, it should be pointed that there is a phase difference between the motion of the affected side and the unaffected side, naturally, In other words, follower 1 first synchronize to the leader's motion trajectory and then follower 2 is expected to track to the leader's trajectory motion after half gait cycle interval.

To guarantee walking assistance control performance, on the basis of LFMAS, the information interaction scheme should be designed for both lower extremities and patient's legs, which means that the information/data (LLE's state and control signal) can be transmitted among them. To this end, the following gives an information exchange rule to describe the evolution of the agents' communication.

- Information Evolution Rule: The information update for follower agent *i* (*i* = 1, 2) includes combining its own information with those received from its neighbors, and Leader can transmit its information to Follower. Assume that each agent has a weight vector *a*_*i*_ = [*a*_*ij*_], in which each element *a*_*ij*_ represents that agent *i* assigns to the information obtained from a neighboring agent *j*. Figure 1B denotes the communication topology network between agents where arrows indicate the direction of information flow.
- Weight Rule: Let N(i) be the neighbors set of the *ith* Follower agent. For arbitrary *i* ∈ {1, 2}, if j∈N(i), *a*_*ij*_ > 0; if j∉N(i), *a*_*ij*_ = 0. Let $\sum_{j\in N\left(i\right)}a_{ij}=d_{i}$ be the sum of the neighbors' weights for agent *i*.

### 2.2. Dynamics Model of LLE System

In this paper, the dynamics of the LLE system is described as a second-order nonlinear mechanical system (i.e., Euler-lagrange system). Therefore, the dynamics of the both lower extremities, i.e., follower 1 (*i* = 1) and follower 2 (*i* = 2) of the exoskeleton are described as follows:

(1) $$\begin{array}{r} H_{i}\left(q_{i}\right)\ddot{q_{i}}+C_{i}\left(q_{i},\dot{q_{i}}\right)\dot{q_{i}}+G_{i}\left(q_{i}\right)=\tau_{i},\;i=1,2 \end{array}$$

where $q_{i}={\left(q_{ih},q_{ik}\right)}^{⊤}\in R^{2}$ denotes the joints' angle of the LLE, *q*_*ih*_ and *q*_*ik*_ represent the hip joint and knee joint, respectively. *H*_*i*_(*q*_*i*_) denotes inertia matrix, $C_{i}\left(q_{i},\dot{q_{i}}\right)$ represents the centripetal and coriolis matrix. *G*_*i*_(*q*_*i*_) denotes the gravitation term, $\tau_{i}={\left(\tau_{iu},\tau_{id}\right)}^{⊤}$ are the input torques generated by up and down motors for hip and knee joint. Further, we can rewrite Equation (1) as a state-space form:

$$\begin{array}{l} \left[\begin{array}{c} {\dot{q}}_{i}\\ {\ddot{q}}_{i} \end{array}\right]=\left[\begin{array}{c} {\dot{q}}_{i}\\ -{H_{i}}^{-1}\left(C_{i}{\dot{q}}_{i}+G_{i}\right) \end{array}\right]+\left[\begin{array}{c} 0\\ {H_{i}}^{-1} \end{array}\right]\tau_{i} \end{array}$$

or equivalently,

(2) $$\begin{array}{r} {\dot{\eta }}_{i}\left(t\right)=f_{i}\left(\eta_{i}\left(t\right)\right)+g_{i}\left(\eta_{i}\left(t\right)\right)u_{i}, \end{array}$$

where $\eta_{i}\left(t\right)={\left[q_{i}^{⊤},{\dot{q}}_{i}^{⊤}\right]}^{⊤}$, $g_{i}\left(\eta_{i}\left(t\right)\right)=\left[\begin{array}{c} \text{0}\\ {H_{i}}^{-1} \end{array}\right]$, $f_{i}\left(\eta_{i}\left(t\right)\right)=\left[\begin{array}{cc} \text{0} & I\\ \text{0} & -{H_{i}}^{-1}C_{i} \end{array}\right]\eta_{i}\left(t\right)+\left[\begin{array}{c} \text{0}\\ -{H_{i}}^{-1}G_{i} \end{array}\right]$, τ_*i*_ = *u*_*i*_.

The dynamic of the leader (the motion trajectory of patient's unaffected leg) is given by:

(3) $$\begin{array}{r} {\dot{\eta }}_{r}\left(t\right)=f\left(\eta_{r}\left(t\right)\right), \end{array}$$

where η_*r*_(*t*) indicates the joint angle collected from human via an IMU sensors matched on the pilot's leg.

#### 2.2.1. Design Objective

The goal is to generate the controller strategy *u*_*i*_ to ensure the trajectory η_*i*_(*t*) generated by Equation (2) can track the trajectory η_*r*_(*t*) in Equation (3). That is, it is desired to make the following tracking error index go to zero:

(4) $$\begin{array}{r} \underset{t\to \infty }{\lim}∥\eta_{i}\left(t\right)-\eta_{r}\left(t\right)∥=0. \end{array}$$

In order to achieve control objective, the local neighbor tracking errors of dynamics (2) for follower *i* are defined as

(5) $$\begin{array}{r} \xi_{i}\left(t\right)=\underset{j\in N\left(i\right)}{\sum }a_{ij}\left(\eta_{i}\left(t\right)-\eta_{j}\left(t\right)\right)+c_{i}\left(\eta_{i}\left(t\right)-\eta_{r}\left(t\right)\right), \end{array}$$

where N(i) and *a*_*ij*_ have been defined in section 2.1. *c*_*i*_ > 0 denotes the pinning gain, which means agent *i* can obtain the Leader's information.

Taking the derivation of Equation (5), combining Equation (2) and Equation (3), the dynamics of the tracking errors are written as

(6) $$\begin{array}{r} \begin{array}{c} {\dot{\xi }}_{i}\left(t\right)=f_{\xi_{i}}+\left(d_{i}+c_{i}\right)g_{i}\left(t\right)u_{i}\left(t\right)-\underset{j\in N\left(i\right)}{\sum }a_{ij}g_{j}\left(t\right)u_{j}\left(t\right), \end{array} \end{array}$$

where $f_{\xi_{i}}\left(t\right)=\sum_{j\in N\left(i\right)}a_{ij}\left(f_{i}-f_{j}\right)+c_{i}\left(f_{i}-f\right)$, *d*_*i*_ indicates the sum of the weights of the *i*^*th*^ follower agent.

## 3. Policy Iteration Based Controller

Based on the system modeling and problem formulation, in this section, the walking assistance control problem will be transformed to an optimal control problem by introducing local cost functions and using optimization theories. Then, the state-of-the-art algorithm called policy iteration (PI) is proposed to obtain the solution to the coupled Hamilton-Jacobi-Bellman (HJB) equation, and thus the optimal controller $u_{i}^{*}\left(t\right)$ is obtained for solving walking assistance problem.

From the perspective of optimal control (Vamvoudakis and Lewis, 2010) and inspired by RL methods (Mnih et al., 2015, 2016; Sutton and Barto, 2018), we use a local cost function to assess the long-term learning and control performance, which is defined as follows:

(7) $$\begin{array}{c} V_{i}(\xi_{i}(t))=\int_{t}^{\infty }r_{i}(\xi_{i}(s),u_{i}(s),u_{\left(j\right)}(s))ds, \end{array}$$

where *u*_(*j*)_(*t*) denotes the neighbors' control of Follower agent *i*, and $r_{i}\left(\xi_{i}\left(t\right),u_{i}\left(t\right),u_{\left(j\right)}\left(t\right)\right)=\xi_{i}^{⊤}\left(t\right)Q_{ii}\xi_{i}\left(t\right)+u_{i}^{⊤}\left(t\right)P_{ii}u_{i}\left(t\right)+\sum_{j\in N\left(i\right)}u_{i}^{⊤}\left(t\right)S_{ij}u_{\left(j\right)}\left(t\right)$ is the reward function, where the *Q*_*ii*_ > 0, *P*_*ii*_ > 0 and *S*_*ij*_ > 0 are symmetric positive definite weighting matrices, respectively. For the notation simplification, we set *r*_*i*_(ξ_*i*_(*t*), *u*_*i*_(*t*), *u*_(*j*)_(*t*)) = *r*_*i*_(ξ_*i*_(*t*), *u*_*i*_(*t*)).

Till now, the walking assistance control problem is transformed into an optimal control problem, which aims to design a distributed controller to guarantee the *Design Objective* as well as minimizing the local cost function (Equation 7).

Further, the Hamilton function is represented as

(8) $$\begin{array}{r} H_{i}(\xi_{i}\left(t\right),u_{i}\left(t\right),V_{i}\left(\xi_{i}\left(t\right)\right))=r_{i}\left(\xi_{i}\left(t\right),u_{i}\left(t\right)\right)+\nabla V_{\xi }^{⊤}{\dot{\xi }}_{i}\left(t\right), \end{array}$$

where *V*_*i*_(0) = 0, ∇*V*_ξ_ = ∂*V*_*i*_(ξ_*i*_(*t*))/∂ξ_*i*_(*t*) is a partial differential part.

Using the stationary condition for Equation (8), i.e., let ∂*H*_*i*_(*t*)/∂*u*_*i*_(*t*) = 0, the optimal controller $u_{i}^{*}\left(t\right)$ is obtained as

(9) $$\begin{array}{r} u_{i}^{*}\left(t\right)=-\frac{1}{2}\left(d_{i}+c_{i}\right)P_{ii}^{-1}g_{i}^{⊤}\left(t\right)\nabla V_{\xi }. \end{array}$$

The optimal cost function $V_{i}^{*}\left(\xi_{i}\left(t\right)\right)$ satisfies the following coupled Hamilton-Jacobi-Bellman (HJB) equation:

(10) $$\begin{array}{r} H_{i}(\xi_{i}\left(t\right),u_{i}^{*}\left(t\right),V_{i}^{*}\left(\xi \left(t\right)\right))=r_{i}\left(\xi_{i}\left(t\right),u_{i}^{*}\left(t\right)\right)+\nabla V_{\xi }^{*⊤}{\dot{\xi }}_{i}\left(t\right)=0. \end{array}$$

Since the coupled HJB equation Equation (10) exists the nonlinear item and partial differential part, which makes it hard to be solved analytically. Therefore, the PI algorithm (Liu and Wei, 2014; Wang et al., 2014), is introduced to approximate the HJB equation and cope with this issue by a successive iteration way.

Let $u_{i}^{l}\left(t\right)$ and $V_{i}^{l}\left(\xi_{i}\left(t\right)\right)$ represent iterative control and iterative Q-function, respectively, with *l* is iteration index. There are two components in PI algorithm, one is *policy evaluation* and the other is *policy improvement*. The detailed iterative performing process can be summarized as follows:

*PI Algorithm*: Start with admissible initial control $u_{i}^{0}$.

Step 1. *Policy Evaluation*: Given the control policy $u_{i}^{l}$, solve for value function $V_{i}^{l}\left(\xi \left(t\right)\right)$ by
  (11) $$\begin{array}{r} H_{i}(\xi_{i}\left(t\right),u_{i}^{l}\left(t\right),V_{i}^{l}\left(\xi \left(t\right)\right))=r_{i}\left(\xi_{i}\left(t\right),u_{i}^{l}\left(t\right)\right)+\nabla V_{\xi }^{l⊤}{\dot{\xi }}_{i}\left(t\right)=0. \end{array}$$
Step 2. *Policy Improvement*: Compute the control law $u_{i}^{l}$ by
  (12) $$\begin{array}{r} u_{i}^{l+1}\left(t\right)=-\frac{1}{2}\left(d_{i}+c_{i}\right)P_{ii}^{-1}g_{i}^{⊤}\left(t\right)\nabla V_{\xi }^{l}. \end{array}$$
Step 3. If $\|V_{i}^{l}\left(\xi_{i}\right)-V_{i}^{l-1}\left(\xi_{i}\right)\|\leq \epsilon$ (ϵ is a small positive constant), end. Else, let *l* = *l* + 1, go to step 1.

The PI algorithm is an effective method to solve the various optimal control problems. It has been proved that the iterative cost function and the iterative control strategy in PI will converge to the optimal values $V_{i}^{⋆}\left(t\right)$ and $u_{i}^{⋆}\left(t\right)$ through iterations (Peng et al., 2019, 2020).

It is worth noting from the above algorithm that the PI algorithm requires the knowledge of system models, i.e., *g*_*i*_(*t*) exists in the controller (Equation 12). In this sense, system identification is needed normally (Ghan and Kazerooni, 2006), but it is not suitable for the practical exoskeleton system with different hemiplegic patient. Since for different wearers/patients, the identification process needs to be reconstructed. To overcome this difficulty, the following section will present a data-driven adaptive control strategy with an online-learning fashion. It should be emphasized that this method avoids needing the knowledge of the accurate system dynamics, and no system identification is introduced.

## 4. Implementation of Controller Design

In this section, we will present the DDOC algorithm base on PI algorithm to achieve online-learning-based control and better adaptive performance for different patients via a neural network (NN) framework of RL called AC/NN. In the AC/NN, actor network is used to approximate controller and critic network is introduced to estimate cost function online, respectively. The detailed descriptions are given as follows.

### 4.1. The Critic NN Modular

First, the critic networks are adopted to approximate the cost function *V*_*i*_(*t*) as follows:

(13) $$\begin{array}{r} {\hat{V}}_{i}\left(t\right)={ŵ}_{ci}^{⊤}\left(t\right)\varphi_{ci}\left(z_{ci}\left(t\right)\right), \end{array}$$

where *z*_*ci*_ is an input information of the critic modular and information from ξ_*i*_, *u*_*i*_, and *u*_(*j*)_, φ_*ci*_(*z*_*ci*_) denotes the activation function, and Ŵ_*ci*_ is the weight vector of the critic network modular.

Then, at each time step, the Hamilton function (8) can be approximated as follows:

(14) $$\begin{array}{l} {𝔢}_{ci}\left(t\right)=\int_{t}^{t+\Delta T}r_{i}\left(\xi_{i},u_{i}\right)ds+{\hat{V}}_{i}\left(t+\Delta T\right)-{\hat{V}}_{i}\left(t\right)\\ \;=\int_{t}^{t+\Delta T}r_{i}\left(\xi_{i},u_{i}\right)ds+{ŵ}_{ci}^{⊤}(\varphi_{ci}\left(z_{ci}\left(t+\Delta T\right)\right)-\varphi_{ci}\left(z_{ci}\left(t\right)\right)), \end{array}$$

where △*T* > 0 denotes the time interval.

Then, the Equation (14) is utilized to define the approximation error for the critic NNs. Thus, the squared residual error function to be minimized is defined as

(15) $$E_{ci}(t)=\frac{1}{2}\|{𝔢}_{ci}(t){\|}^{2}=\frac{1}{2}{𝔢}_{ci}^{2}(t).$$

Then, by making use of gradient descent based weight update rule (Si and Wang, 2001), the tuning weight law can be adopted as follows

(16) $$\begin{array}{l} {\dot{ŵ}}_{ci}\left(t\right)=-\varrho_{ci}\frac{\partial E_{ci}\left(t\right)}{\partial {𝔢}_{ci}\left(t\right)}\frac{\partial {𝔢}_{ci}\left(t\right)}{\partial {\hat{V}}_{i}\left(t\right)}\frac{\partial {\hat{V}}_{i}\left(t\right)}{\partial {ŵ}_{ci}\left(t\right)}\\ \;=-\varrho_{ci}\varphi_{ci}\left(z_{ci}\right)({ŵ}_{ci}^{⊤}\Delta \varphi_{ci}\left(z_{ci}\right)+\int_{t}^{t+\Delta T}r_{i}\left(\xi_{i},u_{i}\right)ds), \end{array}$$

where Δφ_*ci*_(*z*_*ci*_) = φ_*ci*_(*z*_*ci*_(*t*+▵*T*))−φ_*ci*_(*z*_*ci*_(*t*)), ϱ_*ci*_ is the learning rate of the critic network modular for agent *i*.

### 4.2. The Actor NN Modular

Next, define the actor neural networks, which is employed to estimate the control strategy, as follows:

(17) $$\begin{array}{r} {û}_{i}\left(t\right)={ŵ}_{ai}^{⊤}\left(t\right)\varphi_{ai}\left(z_{ai}\left(t\right)\right), \end{array}$$

where *z*_*ai*_ is an input vector of the actor network including ξ_*i*_ of agent *i*, φ_*ai*_(*z*_*ai*_) denotes the activation function, and ŵ_*ai*_ is the weight matrix.

Then, in order to obtain the desired approximation optimal controller to minimize the cost function ${\hat{V}}_{i}$, the error function of the actor network is defined as

(18) $$\begin{array}{r} {𝔢}_{ai}\left(t\right)={\hat{V}}_{i}\left(\xi_{i}\left(t\right)\right)-U_{obj}, \end{array}$$

where $U_{obj}$ is the ultimate objective function. From perspective of the RL, the value of the $U_{obj}$ is selected according to different purposes of applications.

The squared residual error function to be minimized in the actor network is given by

(19) $$\begin{array}{r} E_{ai}\left(t\right)=\frac{1}{2}\|{𝔢}_{ci}\left(t\right){\|}^{2}=\frac{1}{2}{𝔢}_{ai}^{2}\left(t\right). \end{array}$$

Similarly, with the aid of the gradient descent rule, the following updating rule for the actor network is obtained

(20) $$\begin{array}{l} {\dot{ŵ}}_{ai}\left(t\right)=-\varrho_{ai}\frac{\partial E_{ai}\left(t\right)}{\partial {𝔢}_{ai}\left(t\right)}\frac{\partial {𝔢}_{ai}\left(t\right)}{\partial {\hat{V}}_{i}\left(t\right)}\frac{\partial {\hat{V}}_{i}\left(t\right)}{\partial z_{ci}\left(t\right)}\frac{\partial z_{ci}\left(t\right)}{\partial {û}_{i}\left(t\right)}\frac{\partial {û}_{i}\left(t\right)}{\partial {ŵ}_{ai}\left(t\right)}\\ \;=-\varrho_{ai}\varphi_{ai}\left(z_{ai}\right){ŵ}_{ci}^{⊤}\nabla \varphi_{ci}\left(z_{ci}\right)\xi_{i}\varphi_{ci}^{⊤}\left(z_{ci}\right){ŵ}_{ci}, \end{array}$$

where ξ_*i*_ = ∂*z*_*ci*_/∂û_*i*_, ∇φ_*ci*_(*z*_*ci*_) = ∂φ_*ci*_(*z*_*ci*_)/∂*z*_*ci*_ and ϱ_*ai*_ is a learning rate of the actor NN for agent *i*.

The procedure of the data-driven adaptive control strategy is presented in Algorithm 1. It should be noted that only the measured system data, i.e., ξ_*i*_ and *u*_*i*_ are required in the design of the DDOC algorithm. Thus, this method is a data-driven/model-free approach, which improves the potential application of the proposed control method in real systems.

**Algorithm 1:** Optimal Walking Assistance Control Algorithm.

1: **Initialization**
2: Initialize the values of critic weight ŵ_*ci*_(0) and actor weight ŵ_*ai*_(0);
3: Set the learning rates of the critic network and actor network to be ρ_*ai*_ and ρ_*ci*_;
4: Choose a sufficiently small computation precision ϵ;
5: Let *Q*_*ii*_, *P*_*ii*_ and *S*_*ij*_ be positive definite weighting matrices;
6: **repeat**
7: Calculate the actor network to estimate the control strategy û_*i*_ ← (17);
8: Calculate the critic network to estimate the cost function ${\hat{V}}_{i}\leftarrow$ (13);
9: According to the available system data *q*_*i*_ and *q*_*r*_, compute the error ξ_*i*_ ← (5);
10: Calculate the objective function *E*_*ci*_;
11: Update the weights in the critic NNs using ŵ_*ci*_(*t*) ← (16);
12: Calculate the objective function *E*_*ai*_;
13: Update the weights in the actor NNs using ŵ_*ai*_(*t*) ← (20);
14: **until** $\|{ŵ}_{ci}^{\prime }-{ŵ}_{ci}\|\leq \epsilon$ (ŵ_*ci*_ and ${ŵ}_{ci}^{\prime }$ denote the weight of the current time and previous time);

It is noted that in the neural network based approximated structure, some common forms of the activation functions are polynomial functions, tanh functions, sigmoid functions, and so on. Further, we found that the appropriate selection of activation function is very important, which leads to the different size of the NN weight parameters. In this paper, the selection of activation function has the same dimension as the input data. These settings can reduce a huge computation burden for implementation.

## 5. Numerical Simulation

In this section, a *2-DOF manipulator* system in simulation scenario is first carried out to validate the effectiveness of the proposed data-driven control strategy.

### 5.1. Dynamic Model of 2-DOF System

For simulation, the simulation environment is set up in Simulink-Matlab. The dynamics of the two followers are the same as Equation (1), where the system matrices are given as follows: $H_{i}=\left[\begin{array}{cc} m_{i1}+m_{i2}+2m_{i3}\cos(q_{i2}) & m_{i2}+m_{i3}\cos(q_{i2})\\ m_{i2}+m_{i3}\cos(q_{i2}) & m_{i2} \end{array}\right]$, $C_{i}=\left[\begin{array}{cc} -m_{i3}{\dot{q}}_{i2}\sin(q_{i2}) & -m_{i3}({\dot{q}}_{i1})+q_{i2}\sin(q_{i2})\\ -m_{i3}{\dot{q}}_{i1} & 0 \end{array}\right]$ and the $G_{i}=\left[\begin{array}{c} m_{i4}g\cos(q_{i1})+m_{i5}g\cos(q_{i1}+q_{i2})\\ m_{i5}g\cos(q_{i1}+q_{i2}) \end{array}\right]$, τ_*i*_ = [τ_*i*1_, τ_*i*2_]^⊤^, *m*_*ip*_ (*p* = 1, 2, 3, 4, 5) are the masses. Note that, in simulation case, the given dynamic system can be used to product system data needed in DDOC algorithm.

The leader system (desired trajectories) is expressed by

(21) $$\begin{array}{r} q_{r}=\left[\begin{array}{c} q_{1r}\\ q_{2r} \end{array}\right]=\left[\begin{array}{c} 0.5cos\left(t\right)+0.2sin\left(3t\right)\\ 0.3cos\left(3t\right)-0.5sin\left(2t\right) \end{array}\right]. \end{array}$$

We select the structure of the AC/NN as 3-layers back propagation (BP) NN (Goh, 1995). The initial values of critic NN weights and actor NN weights are set to be zero, and setting the value of the computation precision as ϵ = 10^−5^. The weight learning rates of the actor network and the critic network are chosen as ρ_*ai*_ = 0.03, ρ_*ci*_ = 0.06. The activation functions φ_*ai*_ and φ_*ci*_ are selected as the hyperbolic tangent functions, i.e., tanh(*x*) = (*e*^*x*^ − *e*^−*x*^)/(*e*^*x*^ + *e*^−*x*^).

### 5.2. Simulation Results and Analysis

**Figure 2:**
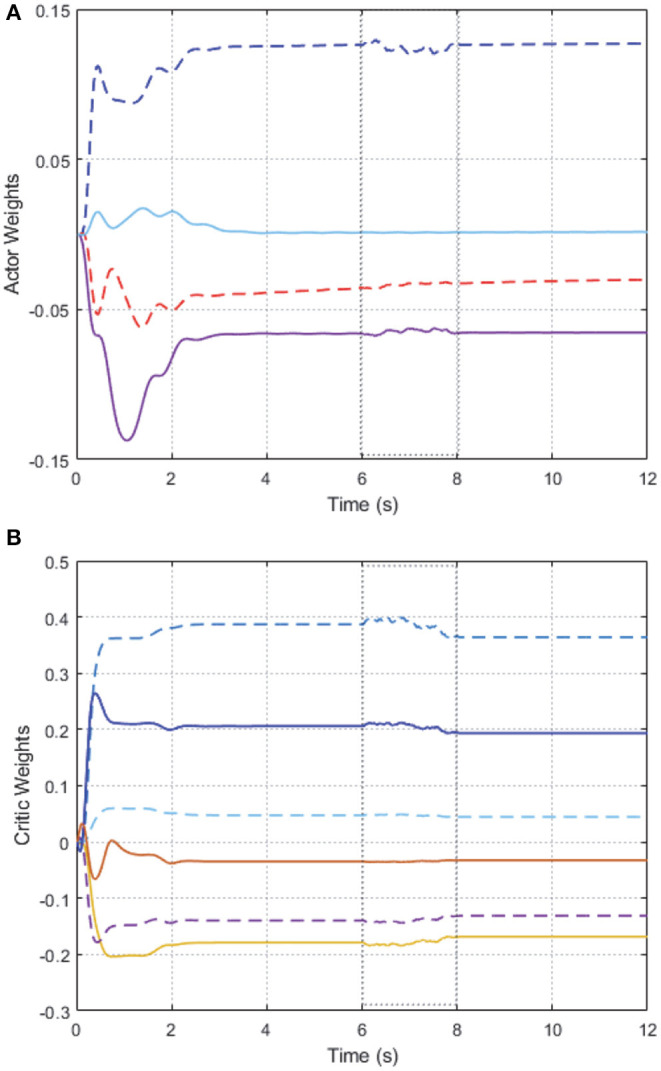
Convergence of the AC/NN weights on 2-DOF simulation platform. **(A)** Actor network. **(B)** Critic network.

**Figure 3:**
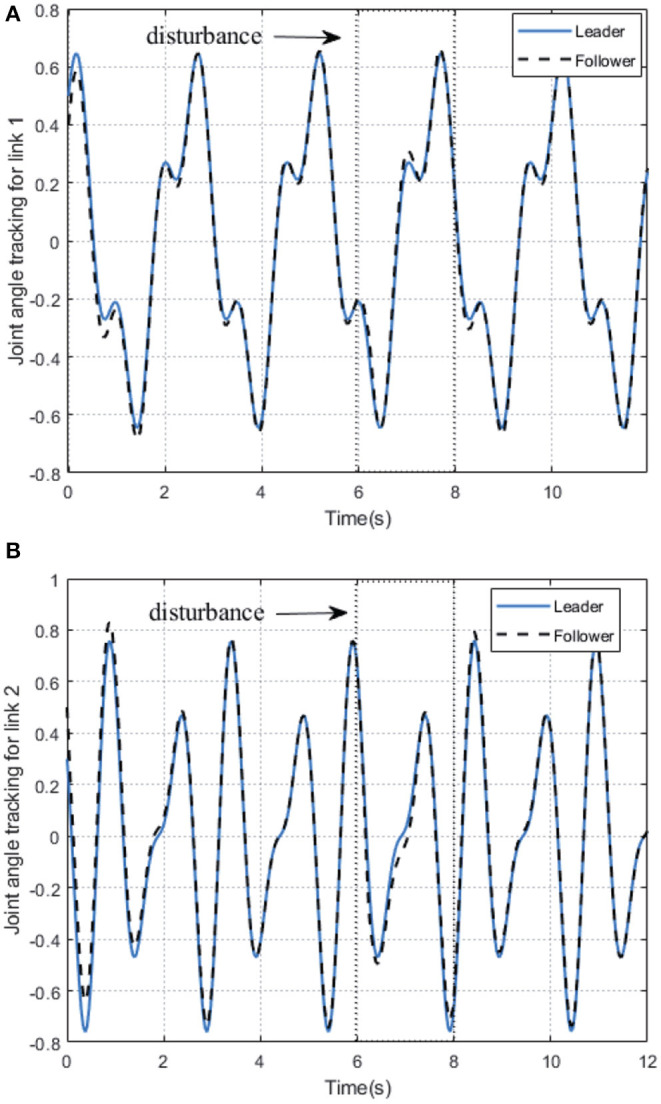
The trajectories tracking performance of joint angle of follower 1 on 2-DOF simulation platform. **(A)** Link 1. **(B)** Link 2.

As shown in Figure 2, we can see that after 2 s learning process, the critic NN weights and the actor NN weights are convergent, and thus the optimal weights parameters are obtained. Therefore, the approximate optimal controller can be obtained in Equation (17). On the basis of the optimal controller, the trajectory of joint angles *q*_1_ = (*q*_11_, *q*_12_)^⊤^ of follower 1 achieves a good tracking performance to the leader at 3 s < *t* < 6 s, which is illustrated in Figure 3.

In order to further verify the performance of adaption against uncertainty, we add some disturbance signal (white noise) to the system at *t* ∈ [6, 7] s. In Figure 2, the AC/NN weights are retrained for learning again adaptively until converge from *t* = 6 s to *t* = 8 s, and thus the optimal controller has been modified correspondingly. With the help of the modified optimal controller, from Figure 3, it is seen that joint angle trajectories of two links of follower 1 are synchronized with the leader again quickly after *t* = 8 s. These simulation results illustrate the better control performance of the proposed DDOC algorithm, which has ability to respond to disturbances online in the system operation. It is proved that our proposed control method has good robustness against uncertainties.

## 6. Experiments on a Real LLE System

In this section, to further verify the control performance of the proposed data-driven control strategy, *walking assistance* experiments on an actual LLE system are performed.

### 6.1. Experimental Setup

**Figure 4:**
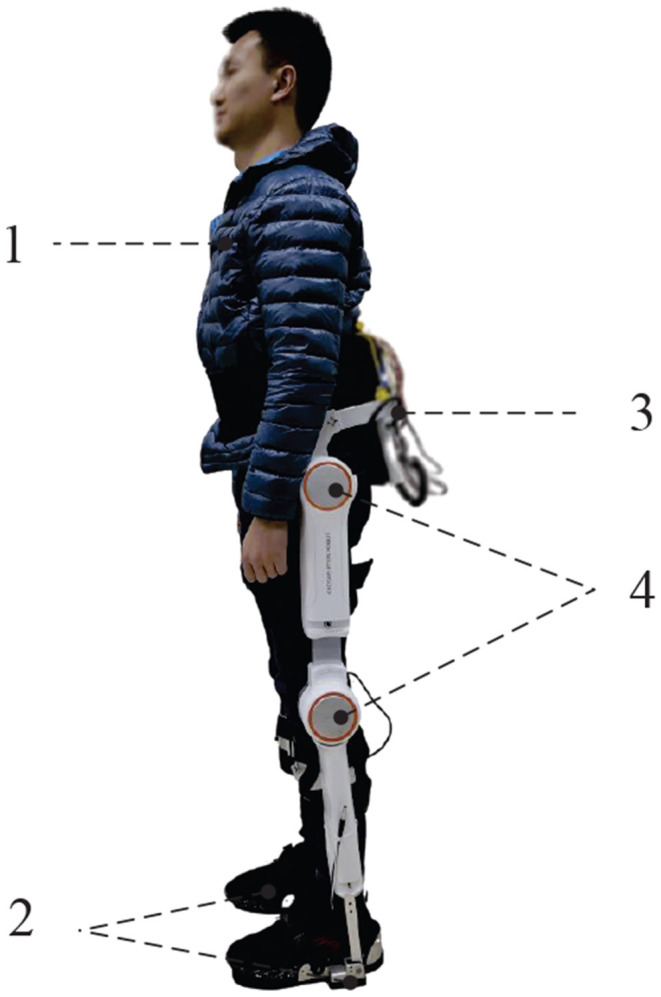
The LLE system called AIDER for hemiplegic patient. 1. The subject/wearer; 2. Smart shoes with plantar pressure sensors inside; 3. The load backpack with embedded computer, IMU and power unit; 4. Active joints with node controllers (hip joints and knee joints).

To demonstrate the effectiveness and adaptability of the proposed control strategy, a practical LLE system, called AIDER, which is shown in Figure 4, is designed for walking assistance case to help hemiplegia. A distributed control system is embedded in AIDER which consists of a main controller and four node controllers. The main controller is placed on the backpack to compute the control algorithm. Node controllers are fixed near by the corresponding active joints position of LLE robot which aims to receive sensor data and execute control commands according to the main controller.

During the experiments on the AIDER system, three healthy subjects (1, 2, 3) with different heights (165, 176, 180 cm) are selected to participate this experiment and operate the wearable LLE robot. All wearers are simulated as hemiplegic patients, and the right legs of the subjects are simulated as the affected leg. In the walking assistance task for all wearers, each wearer is asked to walk for 50 s via the AIDER system. All the pilot's walking speed is varying from 0.1 to 0.4*m*/*s*. Further, the AIDER is equipped with accelerometer and the wearable sensory system for measuring system data.

For the implementation of the proposed data-driven control strategy on the AIDER system. Note that the proposed data-driven control strategy DDOC has a learning process using the online system data at the beginning, which aims to adapt different subjects. After the learning stage, the optimal control policies can be obtained, and then walking assistance can be realized for the LLE system with pilots. We choose the AC/NN as 3-layer Back propagation (BP) NNs structure (Goh, 1995), that is, input layer, hidden layer and output layer. The initial values of weights ŵ_*ci*_ and ŵ_*ai*_ of the critic and actor are all set to be zero, and the activation functions φ_*ai*_ and φ_*ci*_ are chosen as hyperbolic tangent functions tanh(*x*) = (*e*^*x*^ − *e*^−*x*^)/(*e*^*x*^ + *e*^−*x*^). The learning rates are the same as in the simulation, that is ρ_*ai*_ = 0.03, ρ_*ci*_ = 0.06.

### 6.2. Experimental Results and Discussions

**Figure 5:**
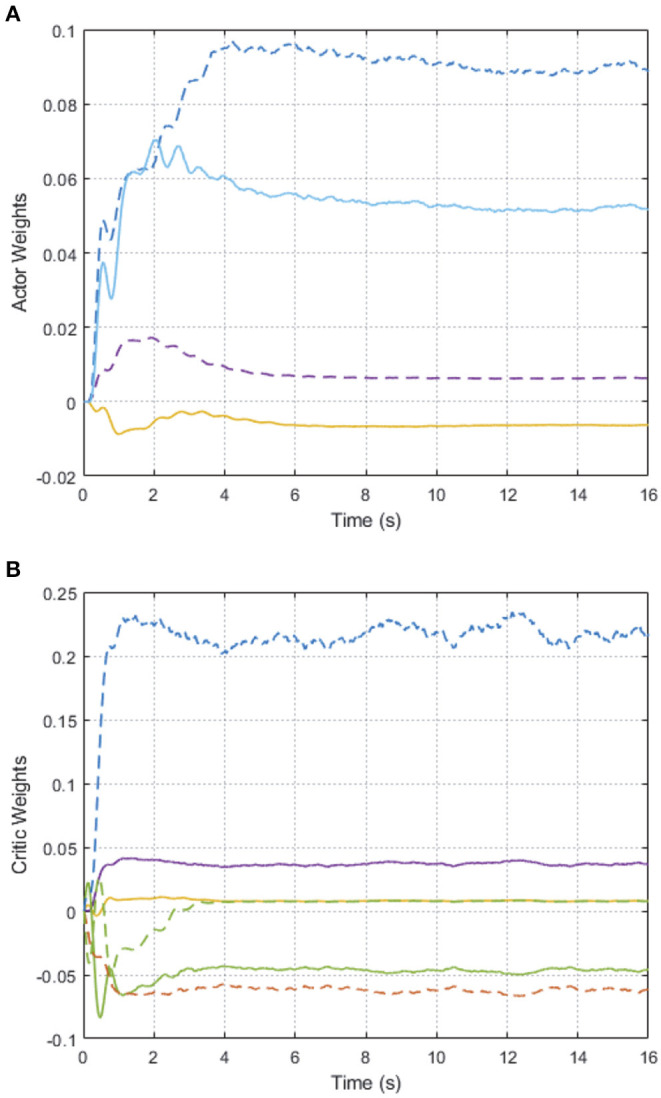
The trajectories of the AC/NN weights for AIDER with subject 1 in the experiment: **(A)** Actor weights. **(B)** Critic weights.

**Figure 6:**
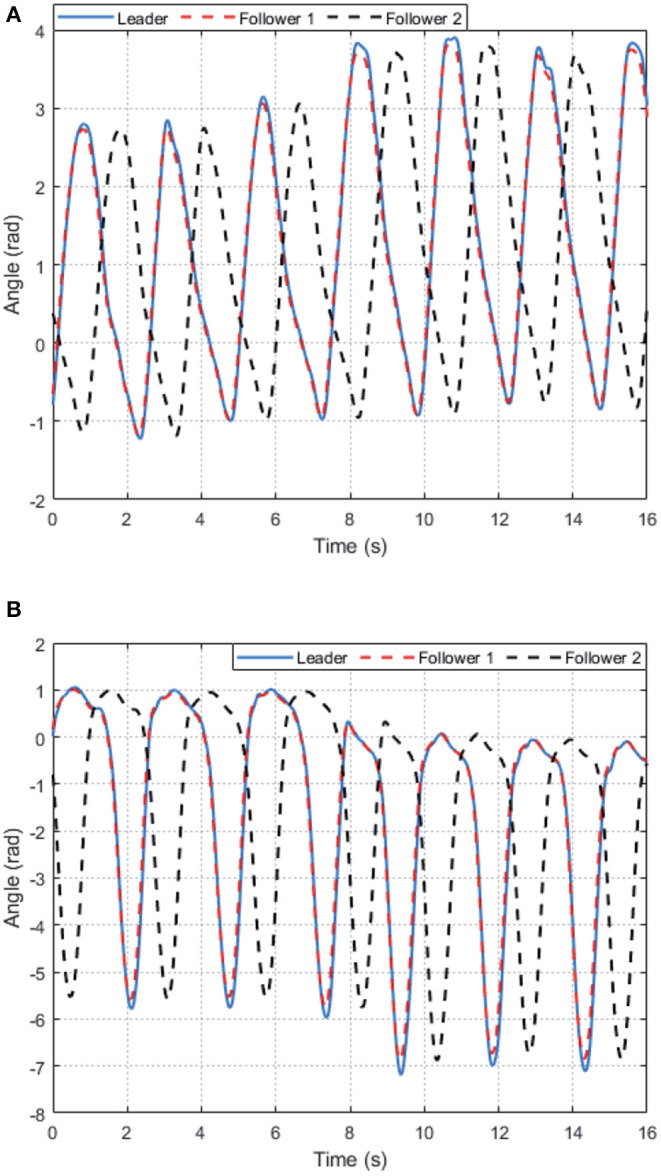
The tracking control performance performance of the proposed DDOC strategy on AIDER with subject 1 in the experiment: **(A)** Hip joint's angle. **(B)** Knee joint's angle.

For participant 1, from Figures 5A,B, we can see that, after about 5 s training, the weights of AC/NN are bounded convergent, i.e., uniformly ultimately bounded because of the disturbances and uncertainties always exist in LLE system. The tracking performance of the hip joint and knee joint for the LLE system with wearer 1 is depicted in Figures 6A,B, which states that with the help of the learned optimal control policies, the hip joint and knee joint of two limbs of the exoskeleton (follower 1, 2) can achieve synchronization with the desired (leader's) motion trajectories. Moreover, it is noted that there are different walking motion patterns in the procedure of walking, which means our proposed method has capability of adapting different gait patterns. It should be pointed out that the affected side of wearer with exoskeleton's side (follower 2) has a half gait cycle delay to the side which has walking ability (leader), which is marked with blue dashed line as shown in Figure 6. In summary, the experimental results illustrate the effectiveness of the proposed DDOC approach for walking assistance of the exoskeleton with different pilots.

## 7. Conclusions

In this paper, a DDOC control strategy has been proposed for a lower exoskeleton system to assist hemiplegic patient walking. A LFMAS structure has been established to model the interaction relation among LLE system and hemiplegic individual. The walking assistance problem has been transformed to an optimal control problem. The PI algorithm has been introduced to obtain optimal assistance controller. On the basis of the PI algorithm, in order to adapt different patients, the AC/NN framework has been presented for the implementation of the proposed approach in an online-learning manner. It highlights that the controller design only relays on the measured system data, rather than the accurate system model. Finally, we have successfully validated the proposed method on two situations: *2-DOF manipulator* in simulation environment and *walking assistance* experiment on a real LLE system called AIDER. Experimental results have confirmed the effectiveness of the proposed control method. In the future, we will focus on more practical control issues, and consider the RL-based controller designs for exoskeleton system with actuator faults and input time-delay.

## Data Availability Statement

The original contributions presented in the study are included in the article/supplementary materials, further inquiries can be directed to the corresponding author/s.

## Ethics Statement

All procedures performed in studies were approved by Research Ethical Committee of University of Electronic Science and Technology of China. Written informed consent was obtained from the participant for the publication of any potentially identifiable images or data included in this article.

## Author Contributions

ZP designed the control methods, performed the experiments, and drafted the manuscript. RL, RH, and KS participated in the design of the controllers and assisted in the manuscript writing. JH and HC guided writing paper and doing experiments. TY designed procedure of experiments and helped to revise and improve the paper significantly.

## Conflict of Interest

The authors declare that the research was conducted in the absence of any commercial or financial relationships that could be construed as a potential conflict of interest.
